# Texture Parameters Measured by UHF-MRI and CT Scan Provide Information on Bone Quality in Addition to BMD: A Biomechanical Ex Vivo Study

**DOI:** 10.3390/diagnostics12123143

**Published:** 2022-12-13

**Authors:** Paul Knoepflin, Martine Pithioux, David Bendahan, François Poullain, Thomas Le Corroller, Cyprien Fabre, Vanessa Pauly, Maud Creze, Enrico Soldati, Pierre Champsaur, Daphne Guenoun

**Affiliations:** 1 Department of Radiology, Institute for Locomotion, Sainte-Marguerite Hospital, APHM, 13009 Marseille, France; 2 Institute of Movement Sciences (ISM), CNRS, Aix Marseille University, 13005 Marseille, France; 3 Centre de Résonnance Magnétique Biologique et Médicale (CRMBM) UMR 7339, CNRS (Centre National de la Recherche Scientifique), Faculté de Médecine, Aix Marseille University, 13005 Marseille, France; 4 Laboratoire de Santé Publique EA3279, Faculté de Médecine, Aix Marseille University, 13005 Marseille, France; 5 Service de Santé Publique et d’Information Médicale, Hôpital de la Conception, APHM, 13005 Marseille, France; 6 Radiology Department, Bicêtre Hospital, APHP, 94270 Le Kremlin–Bicêtre, France

**Keywords:** femur, texture analysis, fracture risk, osteoporosis, micro architecture

## Abstract

The current definition of osteoporosis includes alteration of bone quality. The assessment of bone quality is improved by the development of new texture analysis softwares. Our objectives were to assess if proximal femoral trabecular bone texture measured in Ultra high field (UHF) 7 Tesla MRI and CT scan were related to biomechanical parameters, and if the combination of texture parameters and areal bone mineral density (aBMD) measured by dual-energy X-ray absorptiometry provided a better prediction of femoral failure than aBMD alone. The aBMD of 16 proximal femur ends from eight cadavers were investigated. Nineteen textural parameters were computed in three regions or volumes of interest for each specimen on UHF MRI and CT scan. Then, the corresponding failure load and failure stress were calculated thanks to mechanical compression test. aBMD was not correlated to failure load (R^2^ = 0.206) and stress (R^2^ = 0.153). The failure load was significantly correlated with ten parameters in the greater trochanter using UHF MRI, and with one parameter in the neck and the greater trochanter using CT scan. Eight parameters in the greater trochanter using UHF MRI combined with aBMD improved the failure load prediction, and seven parameters improved the failure stress prediction. Our results suggest that textural parameters provide additional information on the fracture risk of the proximal femur when aBMD is not contributive.

## 1. Introduction

Osteoporosis is a diffuse skeletal disorder that develops with age and characterized by bone fragility, leading to an increased risk of fracture. In 2000, the estimated number of hip fractures was 1,627,000 worldwide and 620,000 in Europe [1]. These fractures are associated with a significant over-morbidity and over-mortality, i.e., 20 to 30% of patients with hip fractures are expected to die within a year.

Bone strength is routinely evaluated from areal bone mineral density (aBMD) measurements using dual-energy X-ray absorptiometry (DXA) [1,2]. Although aBMD has been recognized to be well correlated with fracture risk by the World Health Organization (WHO), the corresponding sensitivity is questionable given that elderly patients with fractures and aBMD values within the normal range, have been reported: Schuit et al. (2004) reported that 39% of men with a hip fracture and 64% of women were considered as osteoporotic according to the corresponding aBMD values [3].

One could hypothesize that aBMD measurement does not completely capture bone quality, which can be characterized at different length scales. Bone size, cortical thickness and geometry can be assessed at a macroscopic scale [4] whereas trabecular connectivity, trabeculae shape and tissue organization can be measured at a microscopic scale. At a nanoscopic scale, the degree of mineralization, the cellular density and the collagen organization can be assessed [5]. All these determinants are expected to influence bone quality and are not included in the aBMD measurements. These multiple parameters highlight the fact that osteoporosis is a multifactorial process that cannot be captured by a single parameter. Accordingly, it has been suggested that multiple bone quality criteria could facilitate the prediction of fractures: Ollivier et al. found that radiographic bone texture analysis provided accurate discrimination between the femoral heads from the fractured and non-fractured groups, and significantly improved the estimation of the femoral neck fracture risk when combined with BMD [6]. Chang et al. concluded that 7 Tesla Magnetic Resonance Imaging (7T MRI) can detect bone micro architectural deterioration in women with fragility fractures who do not differ by BMD [7].

Several studies are evaluating the performance of quantitative computed tomography (QCT): QCT hip fracture discrimination was not significantly higher than DXA discrimination [8], but if QCT of the hip is performed, the combination of trabecular BMD of the trochanter and of cortical thickness of the neck could improve hip fracture discrimination [9].

Bone microarchitecture can be assessed in vivo using several methods such as high-resolution peripheral QCT (HR pQCT) or high-resolution MRI [10]. It has been reported that texture analyses of bone images could also properly reflect bone 3D microarchitecture. Texture analysis has been previously applied to imaging of other areas of the body such as the brain, liver, cartilage, and of tumors [11].

Texture analysis can provide pixel-wise information related to the image contrast [12]. Interestingly, both changes in trabecular thickness and bone texture have been reported in osteoporotic patients [13,14,15,16]. Despite the increasing use of texture analysis in radiology research, there are only few studies assessing texture analysis applied to CT or MRI images for the differentiation of normal bone density from osteoporosis.

Such an analysis has never been conducted in ultra-high field (UHF) magnetic resonance imaging (MRI) which could provide a much higher resolution than 3T MRI and a larger signal to noise.

The purpose of the present study was to assess if ex vivo proximal femoral textural parameters recorded at UHF MRI and CT scan are significantly correlated with biomechanical compression testing simulating a quasi-static sideways fall. The secondary objective was to compare these textural parameters with aBMD and to determine whether a combination of parameters could provide a more sensitive index of bone strength.

## 2. Materials and Methods

### 2.1. Femoral Specimens

Sixteen proximal femora from eight human donors were obtained at the Anatomy Department within 10 days after death, according to institutional safety and ethics regulations. Fifteen femurs from eight donors (6 women and 2 men) were included, and one specimen was excluded for technical reasons (it slipped during the mechanical compression test). Mean age of the donors was 81.8 ± 8.8 yrs. (min = 62, max = 91).

Donor consent for research purposes was obtained prior to death. No information was available on the cause of death or previous diseases. All specimens were carefully cleaned of muscle tissue and ligaments. The femoral diaphysis was cut 10 cm below the lesser trochanter to facilitate bone fixation. The specimens were stored at −20 °C. All specimens were kept hydrated with saline and thawed at room temperature for 6 h before testing so that a single defrosting cycle was required. After defrosting, the imaging protocol was performed and then each specimen was compressed to determine the failure load as previously described [16,17].

### 2.2. CT Measurements

Each specimen was scanned using Light Speed VCT 64 (General-Electric Healthcare) with the following parameters: field of view 12 cm, slice thickness 0.625, interval 0.625 mm, matrix = 512 × 512, tube current mA 365, tube potential kV 120. The voxel size was 0.625 mm^3^. A high-resolution kernel (B60) was used.

Two standard CT quality phantom were used: phantoms CIRS simulating trabecular bone (800 mg/cc) and cortical bone (1750 mg/cc).

### 2.3. DXA Measurements

Specimens were positioned similarly to what is conventionally done for in vivo examination; with a mild internal rotation. They were placed in a vessel filled with tap water up to 15 cm in height to simulate soft tissue [17]. DXA measurements were performed with a Lunar iDXA Scanner (GE/Lunar; GE Medical Systems, Milwaukee, WI, USA). The total proximal aBMD was computed and used for statistical analysis.

### 2.4. MRI Measurements

Each specimen was placed in a rectangular plastic box (Huenersdorff GmbH, Ludwigsburg, Germany; length: 250 mm, width: 100 mm, height: 94 mm) filled with one liter and a half of saline solution (i.e., sodium chloride, 9 g·L^−1^).

All specimens were investigated using an UHF whole body MRI scanner (MAGNETOM 7T, Siemens Medical System, Erlangen, Germany).

After scout images were acquired in the three orthogonal plans, an interactive localized B0 shimming was performed using the second-order shimming procedure provided by the manufacturer.

High-resolution gradient recalled-echo images were acquired in the coronal plane with the following parameters: field of view = 140 × 140 mm^2^; matrix size = 832 × 832; time repetition = 20 ms; echo time = 6 ms; flip angle = 15°; number of repetitions = 3; slice thickness = 0.5 mm and no gap between slices; in-plane pixel size, 0.17 × 0.17 mm. The corresponding acquisition time was 37 min 36 s.

### 2.5. Mechanical Testing

**Figure 1:**
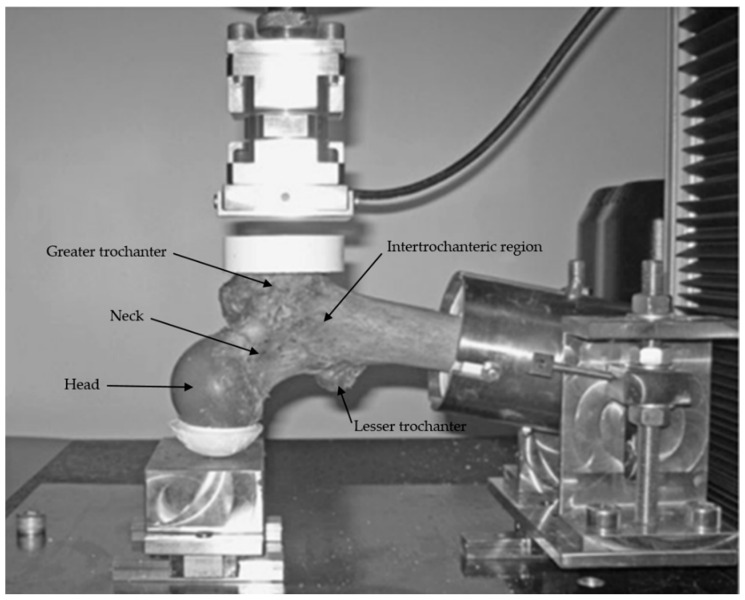
Loading apparatus for mechanical testing of the proximal femur. The orientation of the femur in the loading apparatus was designed to simulate a sideways fall on the greater trochanter.

Each specimen was loaded to failure in a universal testing machine (Instron 5566, Instron, Canton, MA, USA), according to the protocol defined by Le Corroller et al. [16]. The orientation of the femur in the loading apparatus was designed to simulate a sideways fall on the greater trochanter. Specimens were fixed in resin (Epoxy Axon F23) at 15° internal rotation and the femoral shaft was oriented at 10° adduction in the apparatus. The angles were measured with a protractor. Only the femoral shaft was fixed; the femoral head and greater trochanter were free of constraint, so that all of the degrees of freedom are possible at the femoral head, neck and greater trochanter. The load was applied to the greater trochanter through a pad, which simulated a soft tissue cover, and the femoral head was molded with resin to ensure force distribution over a greater surface area (Figure 1). The load was applied at a displacement rate of 10 mm/min. Failure load (in Newtons N) and failure stress (in MegaPascal MPa) were recorded. Failure load was defined as the first local maximum where the load subsequently declined by more than 10%. Then, fractures were visually classified according to clinical criteria (femoral neck, intertrochanteric, subtrochanteric, or isolated greater trochanteric fractures). After fracture, each specimen was scanned again to calculate failure stress: on CT-scan images, on the axial plane, the surface section (in mm^2^) of the fracture site was selected and then the failure stress was calculated:

Failure stress (M P) = Force (N)/Section (in mm²)

### 2.6. Textural Analysis

The texture analysis was performed using the Texture plugin (Olea Sphere v3.0, Olea Medical, La Ciotat, France).

The corresponding analyses were performed in the greater trochanter, intertrochanteric region and femoral neck.

As a preprocessing step of the CT images, air bubbles contained in the volume of interest (VOI) were eliminated using a −500 Hounsfield Unit (HU) threshold and excluded from the calculation of texture parameter to limit bias caused by air bubbles. On CT-images, all elements with a density of less than −500 HU are gaseous elements and therefore correspond to the air bubbles trapped in the bone.

**Figure 2:**
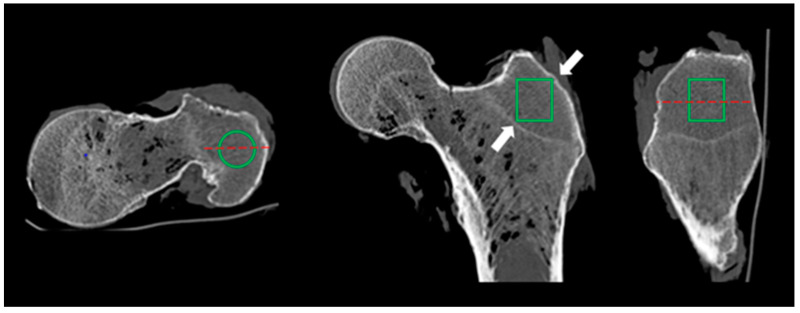
Example of great trochanteric CT cylindric VOI. The VOI is placed from top to bottom in the middle of a line joining external cortical bone and physis line and a line joining anterior and posterior cortex (red dashed lines). The placement avoided the inclusion of cortical bone and physis line in the VOI (white arrows).

The VOI (CT images) has a cylinder-shape (radius = 0.8 cm; length = 1.75 cm). Three VOIs of 3.5 cm^3^ each were assessed for every bone, positioned at the following locations:

− Neck: on the coronal plane, a VOI was placed in the middle of a line passing through the neck axis. This line joined the femoral head physis and a perpendicular line passing through the upper extremity of the greater trochanter physis. The other planes allowed for avoiding cortical bone. The same VOI was used for each specimen.
− Intertrochanteric: on the coronal plane, a VOI was placed at the crossing of the neck and diaphysis axis. The other planes allowed for avoiding cortical bone.
− Greater trochanter: on the axial plane, a VOI was placed in the middle of a line joining the external cortical bone and the physis; on the sagittal plane the middle of a line joining the anterior and posterior cortical bone; on the coronal plane the VOI was placed to avoid cortical bone and physis (Figure 2).

**Figure 3:**
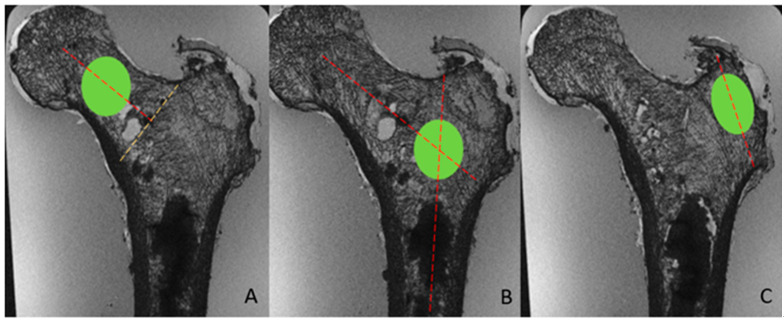
Examples of ROI on MR Image. (**A**): neck placement in the middle of a line passing through the neck axis, joining the femoral head physis and a perpendicular line passing through the intersection of the great trochanter physis and neck cortical bone (yellow dashed line). (**B**): intertrochanteric placement at the intersection of the lines passing through the axis of the diaphysis and the neck. (**C**): greater trochanter placement in the middle of a line corresponding to the vertical axis of the greater trochanter.

For MR images, we used a region of interest (ROI). The ROI has an ovoid-shape, placed on the coronal plane (Figure 3). The ROI dimension was 6500 pixels for the femoral neck, the greater trochanter and the intertrochanteric regions. We did not use a VOI on MRI images because, unlike CT-scan images, voxels were not isometric (0.17 × 0.17 × 0.5 mm). Three ROIs of 6500 pixels each were assessed for every bone, positioned at the following locations:

− Neck: the ROI was placed in the middle of a line passing through the neck axis. This line joined the femoral head physis and a perpendicular line passing through the upper extremity of the great trochanter physis.
− Intertrochanteric: the ROI was placed at the crossing of the neck and diaphysis axis.
− Greater trochanter: the ROI was placed in the middle of a line joining the upper and lower extremities of the greater trochanter (vertical axis).

To assess the inter-rater reliability, textural measurements were performed by two observers on CT and MR images.

First order parameters (Energy, Entropy, Mean and Median) are statistics calculated from the original image values, and do not consider pixel relationships.

Eight textural parameters extracted from the grey level co-occurrence matrices (GLCM) were used: contrast, joint entropy, joint energy, correlation, inverse difference moment, maximum probability, sum average and sum of squares [12]. The GLCM matrix computes the combinations of pixel brightness values (grey levels) in an image. A GLCM of size Ng × Ng describes the second-order joint probability function of an image region constrained by the mask and is defined as P(i,j|δ,θ). The (i,j)th element of this matrix represents the number of times the combination of levels i and j occur in two pixels in the image that are separated by a distance of δ pixels along angle θ. The distance δ from the center voxel is defined as the distance according to the infinity norm. For δ = 1, this results in 2 neighbors for each of 13 angles in 3D (26-connectivity) and for δ = 2 a 98-connectivity (49 unique angles).

Seven textural parameters extracted from the grey level run length matrices (GLRM) were used [18,19]: the short run emphasis, the long run emphasis, the gray level non uniformity, the run length non uniformity, the run percentage, the low gray level run emphasis and the high gray level run emphasis (Table S1). The GLRLM quantifies gray level runs defined as the number of consecutive pixels having the same gray level value. In a gray level run length matrix P(i,j|θ), the (i,j)th element describes the number of runs with gray level i and length j which occurs in the image (ROI) along an angle θ [12].

All of these variables were used in the statistical analysis.

### 2.7. Statistical Analysis

Spearman’s correlation tests were performed between all of the textural parameters and aBMD, failure load, and failure stress. We choose Spearman’s rank correlation coefficient because our group of specimens cannot be considered as a normal population regarding bone status, because there were no data available regarding cause of death or previous illnesses. A multiple linear regression model (with textural parameters adjusted on aBMD values) was performed to assess the independent effect of each textural parameter on failure load and failure stress. The adjusted R^2^ value was computed for each parameter. The adjusted R^2^ represents the amount of variability of failure load or failure stress explained by the model (textural parameter adjusted on aBMD).

Intraclass correlation coefficient (ICC) was used for the reliability analysis for VOI and ROI. ICC values less than 0.5 are indicative of poor reliability, values between 0.5 and 0.75 indicate moderate reliability, values between 0.75 and 0.9 indicate good reliability, and values greater than 0.90 indicate excellent reliability. Statistical significance was determined for *p* values < 0.05. The statistical analyses were performed using RStudio (for Windows, Version 1.1.463).

## 3. Results

During the compression test, a fracture could be detected for a mean failure load and failure stress values of 1433 ± 551 N (min 710; max 2318) and 1.84 ± 0.56 Mpa (min 1.1; max 2.7), respectively (Table 1). We observed six femoral neck and nine intertrochanteric fractures. No sub-trochanteric or isolated greater trochanteric fracture was observed.

The average aBMD value in the neck region was 0.66 ± 0.1 g/cm^2^ (min 0.41; max 0.79) while the overall aBMD value was 0.75 ± 0.11 g/cm^2^ (min 0.5; max 0.88) (Table 1).

Texture parameters were significantly correlated with the failure load (Table 2).

In both, the femoral neck and the intertrochanteric region of the MR images, no significant correlation was found between texture parameters and failure load (Table S2).

A similar analysis was conducted for the CT images and the corresponding results were different.

The GLRM Run length non uniformity was the only parameter significantly correlated with failure load in both the neck (*p* = 0.045) and the greater trochanter (*p* = 0.031) regions (Table S3).

Regarding the correlation with total aBMD, eight texture parameters computed in the greater trochanter region of the MR images and four in the neck region of the CT images were significantly correlated (Table 3 and Table S4).

Eight texture parameters computed in the greater trochanter region of the MR images were correlated with failure stress (Table 4).

In this region, the combination of eight textural parameters for failure load and seven for failure stress, with total aBMD of proximal femur, significantly improved the prediction of failure R^2^ (Table 5 and Table 6).

For example, the multiple regression analysis found that the combinations of aBMD and textural parameters used to explain the femur failure load significantly improved R² from 0.206 for aBMD alone, to an adjusted R^2^ = 0.783 for GLCM Sum Average. More specifically, GLCM Sum Average characteristics was the variable with the largest influence on the R^2^ increase when combined to aBMD: when aBMD was combined with GLCM Sum Average adjR^2^ yielded the value of 0.576 (improvement of 379%).

For failure stress, GLRM Low Gray Level Run Emphasis was the variable with the largest influence on the R^2^ increase when combined with aBMD: adjR^2^ yielded the value of 0.307 (improvement of 301%).

The average ICC was 0.81 ± 0.13 (min: 0.63; max: 0.99).

## 4. Discussion

Our study intended to assess different statistical textural parameters in MR and CT images of proximal femur specimens and to evaluate the relationship with bone strength and bone mineral density.

Eight texture parameters improved fracture risk information: First Order Entropy; GLCM Joint Energy; GLCM Joint Entropy; GLCM Sum Average; GLCM Sum of Squares; GLRM Gray Level Non Uniformity; GLRM Low Gray Level Run Emphasis and GLRM High Gray Level Run Emphasis. First order entropy is calculated based on the distribution of the pixel values in the kernel. It measures the disorder of the kernel values. Grey Level Co-occurrence did not provide any information about the repeating nature of texture. GLCM contains information about the positions of pixels having similar gray level values. GLRM is a set of constant intensity pixels located in a line. Runlength statistics are calculated by counting the number of runs of a given length for each grey level [12] (Table S1). We believe these texture features are reflective of osteoporotic changes in the cancellous bone as they reflect the architectural disorganization of the bone.

The femoral strength (mean failure load values = 1433 ± 551 N) was in accordance with other further studies: Guenoun et al. found a mean failure load at 1238 N and Soldati et al. found a mean failure load at 1733.6 N [17,20]. In these studies, the femoral mechanical compression test was the same as our study with a load applied at a displacement rate of 10 mm/min. However, femoral strength was quite low when compared to the study of Pullkinen et al. that found mean failure load for cervical fractures was 2879 in women and 4079 (load was applied at a rate of 6.6 mm/s) [21]. Dragomir-Daescu et al. demonstrated that sex accounted for a significant difference of >1000 N in femoral strength between women and men, but we did not have such a difference in our study [22].

Surprisingly, aBMD was not correlated to failure load and stress. We chose total aBMD instead of neck aBMD because texture parameters were measured at different sites (neck, intertrochanteric and greater trochanter sites), and because Fractures occurred mainly in the intertrochanteric regions. Intertrochanteric Fractures are common extra-capsular fractures of the proximal femur at the level of the greater and lesser trochanter that are most commonly seen following ground-level falls in the elderly population. This lack of correlation between aBMD and failure load is unusual compared to our previous studies with the same fracture protocol [16,20]. The discrepancy in our results can be an artifact of sampling: we only had six females and two males, and one of the males was 18 years younger than the youngest female. Age may play a role here. However, we can assume that these specimens could also represent cases where aBMD is not sufficient to detect osteoporosis and where texture analysis could provide additional information on bone quality. Indeed, many authors argued that aBMD is a poor predictor of fracture risk: Stone et al. found that only 28% of hip fractures occurred in women with established osteoporosis at baseline [23].

In the present study, in MRI analysis, the greater trochanter was the only region in which texture and failure load variables were significantly correlated. This could be explained by the better image quality in the trochanteric region and because the force was applied there. Our results agree with the study of Chang et al. which found significant results only in the greater trochanter site [24], and the study of Le Corroller and al. who found a better correlation between textural parameters obtained in the greater trochanter by high-resolution X-ray device and mechanical testing [25]. Unlike X-ray based methods, cadaveric bone imaging through MRI is more challenging than in vivo imaging for several reasons [26]. On CT-images air bubbles contained in the volume of interest (VOI) were easily eliminated using a −500 UH threshold but this post -processing was not applicable to MRI. We did not find any post-processing software capable of removing bubbles. Indeed, air that leaks into the marrow space during sample preparation or during the decomposition process creates signal voids that could be misclassified as “bone” signal. In addition, air inclusions cause magnetic susceptibility artifacts leading to artificial broadening of trabecular bone thickness during MRI acquisitions. Air bubbles were more present in inter trochanteric and neck region probably because of the proximity to the cutting section and metabolic degradation processes. We tried to avoid regions with air bubbles for the measurements but some air bubbles may have impacted the results and may explain the lower correlations at these sites.

Our results could have been better if we had done 3D texture analysis on MR images with a VOI, but the ROI was chosen because in MRI images the voxel was not isometric and to avoid air bubbles more easily.

Our results are consistent with other studies demonstrating that the combination of bone quality markers with aBMD improve the prediction of failure [27,28]. Thevenot et al. reported that the combination of first order entropy measured on plain radiographic images and aBMD improved the in vivo discrimination between women with and without fracture [28]. This combination was also one of our significant results in the greater trochanter for both failure load and stress. The study of Chappard et al. showed that the combination of GLCM textural parameters and cortical thickness assessed on plain radiography performed as well as total aBMD alone to predict failure load [29]. Their results, like ours, were better in the greater trochanter.

ICC was good to excellent [30], and for only two parameters (GLCM maximum probability and GLRM Run length non uniformity) the ICC were moderate, but these parameters were not significantly correlated with failure load or stress. These parameters are more sensitive to variations of VOI/ROI placement: GLCM maximum probability is the probability corresponding to the most common grey level cooccurrence in the GLCM and may vary if the pixels selected in the VOI/ROI of the two observers have a different grey level. The GLRM Run length non uniformity assesses the distribution of runs over the run lengths and may vary if the VOI/ROI are placed by the two observers in a region with a different homogeneity.

While many correlations between texture parameters and mechanical testing were found in MRI, ten texture parameters with failure load and eight with failure stress, we do not clearly explain why only one parameter was positive in the CT scan analyses. Initially, we thought we would get better results in CT than in MRI because it is a better exam to evaluate the bone. We can explain this result by the image resolution: the in-plane resolution was better in MRI (0.17 mm), closer to trabecular thickness, than in CT-scan (0.625 mm).

Limitations in our study were the small number of specimens and the presence of gas bubbles that may have altered the textural parameters. Our group of specimens cannot be considered as a normal population regarding bone status because there were no data available regarding cause of death or previous illnesses. The use of paired femurs from a single donor restricts the variance of the results and the statistics were made as if the data were independent. The impact of those statistics is that we lose power.

We acknowledge that the use of frozen/thawed specimens limits application to in vivo proximal femurs. Furthermore, the use of defleshed bones is not always reflective of in vivo measures: even if we used tap water to simulate soft tissues, DXA results may have been altered, as well as mechanical tests.

For textural parameters measurements, we used a ROI on MRI images and a VOI on CT-scan images. Even if we attempted to select the same area of interest in CT and MRI, it probably slightly influenced the results.

Our ICC was moderate to good, in particular for parameters that were significant. Our two lowest ICC values were not for parameters found significant in the multiple analyses.

For cost reason and low availability, 7 tesla MRI is only used in research. However, the significant results found in 7 telsa MRI must now be found in routine 1.5 or 3 T MRI.

## 5. Conclusions

Our study shows which textural parameters among the many available parameters are well correlated with failure load and failure stress. In this sample of femurs where aBMD is not correlated to failure load and stress, combining textural parameters with aBMD improve the prediction of fracture in comparison with aBMD alone. We suggest that when aBMD is not sufficient to detect osteoporosis, texture analysis could provide additional information on bone quality.

## Figures and Tables

**Table 1:** Information on the subjects and femurs. Sex, age, total femur bone mineral density (g/cm^2^), failure load of the femur (N) and failure stress (MPa).

Sex	Age	Femur Side	Total Femur BMD	Failure Load (N)	Failure Stress (MPa)
Women	81	Right	0.849	2075.36	2.39
		Left	0.861	2113.96	2.38
Women	83	Right	0.701	1293.2	2.13
		Left	0.714	1477.2	2.51
Women	83	Right	0.722	2318.69	2.31
		Left	0.651	1524.42	1.55
Women	86	Right	0.735	866.9	1.14
		Left	0.701	excluded	excluded
Women	89	Right	0.508	743	1.1
		Left	0.615	973	1.5
Men	62	Right	0.861	1114	1.33
		Left	0.842	1494	1.86
Men	80	Right	0.884	1760.04	2.12
		Left	0.849	2148.1	2.73
Women	91	Right	0.773	710.58	1.16
		Left	0.731	876.28	1.36

**Table 2:** Correlation between textural parameters and failure load for the greater trochanter in the MR images. In bold and italic, *p*-value under 0.05. (aBMD: areal bone mineral density; GLCM: grey level coocurrence matrix; GLRM: grey level run-length matrix).

Textural Parameters	r	*p* Value
First Order		
Energy	−0.489	(0.066)
Entropy	−0.821	***(0.0002)***
Mean	−0.532	***(0.043)***
Median	−0.625	***(0.014)***
**GLCM**		
Contrast	−0.282	(0.307)
Correlation	−0.182	(0.515)
Joint Energy	0.75	***(0.0019)***
Joint Entropy	−0.7	***(0.0048)***
Inverse Difference Moment	0.228	(0.411)
Maximum Probability	0.489	(0.066)
Sum Average	−0.864	***(<0.0001)***
Sum of Squares	−0.753	***(0.0017)***
**GLRM**		
Short Run Emphasis	−0.26	(0.346)
Long Run Emphasis	0.214	(0.442)
Gray Level Non Uniformity	0.589	***(0.023)***
Run Length Non Uniformity	−0.010	(0.974)
Run Percentage	−0.214	(0.442)
Low Gray Level Run Emphasis	0.846	***(<0.0001)***
High Gray Level Run Emphasis	−0.896	***(<0.0001)***

**Table 3:** Correlation between textural parameters and aBMD for the greater trochanter and neck in the MR images and for the greater trochanter and the neck in the CT images. In bold and italic, *p*-value under 0.05. (GT = greater trochanter). (aBMD: areal bone mineral density; GLCM: grey level coocurrence matrix; GLRM: grey level run-length matrix).

	MRI GT Neck				CT GT Neck			
Textural Parameters	r	*p* Value	r	*p* Value	r	*p* Value	r	*p* Value
First Order								
Energy	−0.137	(0.624)	0.086	(0.761)	0.191	(0.477)	−0.160	(0.552)
Entropy	−0.631	***(0.011)***	−0.259	(0.35)	0.091	(0.737)	−0.368	(0.160)
Mean	−0.116	(0.679)	0.07	(0.805)	0.263	(0.324)	−0.720	***(0.0016)***
Median	−0.134	(0.633)	0.086	(0.761)	0.295	(0.266)	−0.546	***(0.028)***
**GLCM**								
Contrast	−0.135	(0.629)	−0.263	(0.344)	0.059	(0.828)	0.108	(0.687)
Correlation	−0.250	(0.367)	0.114	(0.684)	0.258	(0.335)	−0.764	***(0.0005)***
Joint Energy	0.599	***(0.018)***	0.288	0.298)	−0.081	(0.765)	0.362	(0.167)
Joint Entropy	−0.583	***(0.022)***	−0.327	(0.233)	0.043	(0.875)	−0.288	(0.278)
Inverse Difference Moment	0.180	(0.519)	0.164	(0.558)	−0.103	(0.704)	0.116	(0.667)
Maximum Probability	0.567	***(0.027)***	0.182	(0.515)	−0.277	(0.299)	0.288	(0.278)
Sum Average	−0.533	***(0.04)***	−0.161	(0.566)	0.187	(0.488)	−0.319	(0.227)
Sum of Squares	−0.556	***(0.031)***	−0.218	(0.434)	0.122	(0.652)	−0.379	(0.146)
**GLRM**								
Short Run Emphasis	−0.191	(0.494)	−0.213	(0.446)	0.109	(0.688)	0.081	(0.765)
Long Run Emphasis	0.135	(0.629)	0.164	(0.558)	−0.128	(0.636)	−0.108	(0.687)
Gray Level Non Uniformity	0.332	(0.225)	−0.172	(0.54)	0.085	(0.753)	0.474	(0.063)
Run Length Non Uniformity	−0.071	(0.799)	−0.268	(0.333)	0.182	(0.498)	0.307	(0.246)
Run Percentage	−0.135	(0.629)	−0.172	(0.54)	0.125	(0.644)	0.081	(0.765)
Low Gray Level Run Emphasis	0.533	***(0.040)***	0.086	(0.761)	−0.11	(0.684)	0.522	***(0.037)***
High Gray Level Run Emphasis	−0.529	***(0.042)***	−0.181	(0.519)	0.215	(0.424)	−0.343	(0.193)

**Table 4:** Correlation between textural parameters and failure stress in great trochanter MRI. In bold and italic, *p*-value under 0.05. (GLCM: grey level co-occurencematrix; GLRM: grey level run-length matrix).

Textural Parameters	r	*p*-Value
First Order		
Energy	−0.312	(0.257)
Entropy	−0.692	***(0.004)***
Mean	−0.455	(0.088)
Median	−0.475	(0.073)
**GLCM**		
Contrast	−0.194	(0.487)
Correlation	−0.445	(0.096)
Joint Energy	0.593	***(0.019)***
Joint Entropy	−0.617	***(0.014)***
Inverse Difference Moment	0.122	(0.663)
Maximum Probability	0.668	***(0.006)***
Sum Average	−0.707	***(0.003)***
Sum of Squares	−0.681	***(0.005)***
**GLRM**		
Short Run Emphasis	−0.079	(0.778)
Long Run Emphasis	0.084	(0.764)
Gray Level Non Uniformity	0.371	(0.172)
Run Length Non Uniformity	−0.22	(0.429)
Run Percentage	−0.083	(0.767)
Low Gray Level Run Emphasis	0.725	***(0.002)***
High Gray Level Run Emphasis	−0.718	***(0.002)***

**Table 5:** Multiple regression analysis: combinations of aBMD and MRI textural parameters used to explain the femur failure load (aBMD: areal bone mineral density; GLCM: grey level co-occurrence matrix; GLRM: grey level run-length matrix). In bold and italic, *p*-value under 0.05.

	R²	Adjusted R²	*p*-Value
aBMD Alone	0.2066		
aBMD + MRI Textural parameters			
First Order Entropy		0.569	***(0.0012)***
GLCM Joint Energy		0.431	***(0.0128)***
GLCM Joint Entropy		0.48	***(0.006)***
GLCM Sum Average		0.783	***(<0.0001)***
GLCM Sum of Squares		0.53	***(0.0025)***
GLRM Gray Level Non Uniformity		0.348	***(0.0412)***
GLRM Low Gray Level Run Emphasis		0.761	***(<0.0001)***
GLRM High Gray Level Run Emphasis		0.782	***(<0.0001)***

**Table 6:** Multiple regression analysis: combinations of aBMD and MRI textural parameters used to explain the femur failure stress (aBMD: areal bone mineral density; GLCM: grey level coocurrence matrix; GLRM: grey level run-length matrix). In bold and italic, *p*-value under 0.05.

	R²	Adjusted R²	*p*-Value
aBMD Alone	0.1530		
aBMD + MRI Textural parameters			
First Order Entropy		0.407	***(0.0103)***
GLCM Joint Entropy		0.299	***(0.0444)***
GLCM Sum Average		0.433	***(0.0071)***
GLCM Sum of Squares		0.401	***(0.0113)***
GLRM Low Gray Level Run Emphasis		0.46	***(0.0047)***
GLRM High Gray Level Run Emphasis		0.452	***(0.0054)***

## Data Availability

Data are the property of Assistance Publique Hopitaux de Marseille and Aix-Marseille University. Data are not available on open source but are available on request.

## Supplementary Materials

The following supporting information can be downloaded at: https://www.mdpi.com/article/10.3390/diagnostics12123143/s1. Table S1: textural parameters formulas; Table S2: Correlation between textural parameters and failure load; Table S3: Correlation between textural parameters and failure load for the femoral neck and the great trochanter in CT images; Table S4: Correlation between textural parameters and aBMD.
