# Supplementary material for: Texture Parameters Measured by UHF-MRI and CT Scan Provide Information on Bone Quality in Addition to BMD: A Biomechanical Ex Vivo Study
Source: Diagnostics (Basel). 2022 Dec 13;12(12):3143. doi: 10.3390/diagnostics12123143 (PMC9777398; doi:10.3390/diagnostics12123143)
Supplement: Supplementary file 1 [file diagnostics-12-03143-s001.zip › diagnostics-1940870-supplementary.pdf]

Table S1: textural parameters formulas

ANNEX (from Technical Document Texture Olea Medical):
Textural parameters formulas:
First order:

$$energy = \sum_{i=1}^{N_p} (\mathbf{X}(i) + c)^2 \qquad entropy = -\sum_{i=1}^{N_g} p(i)\log_2(p(i)+\varepsilon) \qquad mean = \frac{1}{N_P}\sum_{i=1}^{N_P} X(i)$$

With:
X be a set of Np voxels included in the ROI/VOI
P(i) be the first order histogram with Ng discrete intensity levels, where Ng is the number
of non-zero bins, equally spaced from 0 with a width defined in the binWidth parameter.
p(i) be the normalized first order histogram and equal to P(i)/Np

Co-occurrence parameters:

$$contrast = \sum_{i=1}^{N_g}\sum_{j=1}^{N_g} (i-j)^2 p(i,j) \qquad correlation = \frac{\sum_{i=1}^{N_g}\sum_{j=1}^{N_g} p(i,j)ij - \mu_x\mu_y}{\sigma_x(i)\,\sigma_y(j)} \qquad joint\ energy = \sum_{i=1}^{N_g}\sum_{j=1}^{N_g} (p(i,j))^2$$

$$joint\ entropy = \sum_{i=1}^{N_g}\sum_{j=1}^{N_g} p(i,j)\log_2(p(i,j)+\varepsilon) \qquad IDM = \sum_{i=1}^{N_g}\sum_{j=1}^{N_g}\frac{p(i,j)}{1+|i-j|^2} \qquad sum\ average = \sum_{k=2}^{2N_g} p_{x+y}(k)k$$

$$maximum\ probalility = max\ (p(i,j)) \qquad sum\ squares = \sum_{i=1}^{N_g}\sum_{j=1}^{N_g}(i-\mu_x)^2 p(i,j)$$

With:
ε be an arbitrarily small positive number (≈2.2×10−16)
P(i,j) be the co-occurence matrix for an arbitrary δ and θ
p(i,j) be the normalized co-occurence matrix and equal to:
Ng be the number of discrete intensity levels in the image
$p_x(i) = \sum_{j=1}^{N_g} P(i,j)$ be the marginal row probabilities
$p_y(j) = \sum_{i=1}^{N_g} P(i,j)$ be the marginal column probabilities

μx be the mean gray level intensity of px and defined as: $\mu_x = \sum_{i=1}^{N_g} p_x(i)\, i$

μy be the mean gray level intensity of py and defined as: $\mu_y = \sum_{j=1}^{N_g} p_y(j)\, j$
σx be the standard deviation of px
σy be the standard deviation of py

Run length parameters:

$$SRE = \frac{\sum_{i=1}^{N_g} \sum_{j=1}^{N_r} \frac{P(i,j|\theta)}{j^2}}{N_z(\theta)}$$

$$LRE = \frac{\sum_{i=1}^{N_g} \sum_{j=1}^{N_r} P(i,j|\theta)\, j^2}{N_z(\theta)}$$

$$GLN = \frac{\sum_{i=1}^{N_g} \left(\sum_{j=1}^{N_r} P(i,j|\theta)\right)^2}{N_z(\theta)}$$

$$RLN = \frac{\sum_{j=1}^{N_r} \left(\sum_{i=1}^{N_g} P(i,j|\theta)\right)^2}{N_z(\theta)}$$

$$RP = \frac{N_z(\theta)}{N_p}$$

$$LGLRE = \frac{\sum_{i=1}^{N_g} \sum_{j=1}^{N_r} \frac{P(i,j|\theta)}{i^2}}{N_z(\theta)}$$

$$HGLRE = \frac{\sum_{i=1}^{N_g} \sum_{j=1}^{N_r} P(i,j|\theta)\, i^2}{N_z(\theta)}$$

With :
Ng be the number of discreet intensity values in the image
Nr be the number of discreet run lengths in the image
Np be the number of voxels in the image

Nz(θ) be the number of runs in the image along angle θ, which is equal to $\sum_{i=1}^{N_g} \sum_{j=1}^{N_r} \frac{P(i,j)}{\theta}$
and 1≤Nz(θ)≤Np
P(i,j|θ) be the run length matrix for an arbitrary direction θ
p(i,j|θ) be the normalized run length matrix, defined as p(i,j|θ)=P(i,j|θ)/Nz(θ)

Table S2: Correlation between textural parameters and failure load

In bold and italic, p-value under 0,05. (GT= great trochanter).

Textural parameters	MRI Neck		MRI GT		MRI Inter-trochanteric		CT Neck		CT GT		CT Inter-trochanteric	
First Order Energy	-0.2	(0.4737)	-0.4892	(0.0665)	-0.1571	(0.5755)	0.0107	(0.9744)	0.3714	(0.1734)	0.1785	(0.5235)
First Order Entropy	-0.175	(0.5320)	-0.8214	***(0.0002)***	-0.0142	(0.9642)	0.025	(0.9336)	-0.0571	(0.8424)	0.1571	(0.5755)
First Order Mean	-0.1678	(0.5492)	-0.5321	***(0.0437)***	-0.2428	(0.3819)	-0.4464	(0.0972)	0.2857	(0.3011)	-0.2392	(0.3892)
First Order Median	-0.1251	(0.6568)	-0.625	***(0.0148)***	-0.2285	(0.4114)	-0.3378	(0.2181)	0.2361	(0.3968)	-0.2392	(0.3892)
Gray Level Co-occurrence Matrix Contrast	0.3107	(0.2591)	-0.2821	(0.3074)	0.3928	(0.1484)	0.2428	(0.3819)	0.0357	(0.9030)	0.3535	(0.1963)
Gray Level Co-occurrence Matrix Correlation	-0.3642	(0.1823)	-0.1821	(0.5150)	-0.1571	(0.5755)	-0.3071	(0.2649)	0.0535	(0.8525)	-0.325	(0.2369)
Gray Level Co-occurrence Matrix Joint Energy	-0.0357	(0.9030)	0.75	***(0.0019)***	-0.225	(0.4189)	-0.0928	(0.7434)	-0.125	(0.6575)	-0.0214	(0.9438)
Gray Level Co-occurrence Matrix Joint Entropy	-0.0571	(0.8424)	-0.7	***(0.0048)***	0.2357	(0.3965)	0.1107	(0.6952)	0.0285	(0.9234)	0.1	(0.7240)
Gray Level Co-occurrence Matrix Inverse Difference Moment	-0.35	(0.2011)	0.2285	(0.4114)	-0.375	(0.1691)	-0.1392	(0.6205)	-0.1678	(0.5492)	-0.1071	(0.7048)
Gray Level Co-occurrence Matrix Maximum Probability	0.1357	(0.6297)	0.4892	(0.0665)	0.0571	(0.8424)	-0.0678	(0.8124)	-0.1571	(0.5755)	0.2535	(0.3607)
Gray Level Co-occurrence Matrix Sum Average	0.1071	(0.7048)	-0.8642	***(<0.0001)***	-0.0357	(0.9030)	-0.1928	(0.4900)	0.05	(0.8625)	0.075	(0.7925)
Gray Level Co-occurrence Matrix Sum of Squares	-0.3142	(0.2535)	-0.7535	***(0.0017)***	-0.0214	(0.9438)	0.0178	(0.9540)	-0.0571	(0.8424)	0.2	(0.4737)
Gray Level Run Length Matrix Short Run Emphasis	0.225	(0.4189)	-0.2607	(0.3469)	0.4107	(0.1296)	0.2142	(0.4420)	0.125	(0.6575)	0.0964	(0.7337)
Gray Level Run Length Matrix Long Run Emphasis	-0.2178	(0.4342)	0.2142	(0.4420)	-0.3285	(0.2316)	-0.2142	(0.4420)	-0.1071	(0.7048)	-0.0285	(0.9234)
Gray Level Run Length Matrix Gray Level Non Uniformity	-0.1785	(0.5235)	0.5892	***(0.0232)***	0.2071	(0.4577)	0.3964	(0.1445)	0.375	(0.1691)	0.2142	(0.4420)
Gray Level Run Length Matrix Run Length Non Uniformity	-0.1857	(0.5066)	-0.0107	(0.9744)	0.1392	(0.6205)	0.5285	***(0.0454)***	0.5642	***(0.0310)***	0.3714	(0.1734)
Gray Level Run Length Matrix Run Percentage	0.2285	(0.4114)	-0.2142	(0.4420)	0.4178	(0.1226)	0.2142	(0.4420)	0.1464	(0.6023)	0.0285	(0.9234)
Gray Level Run Length Matrix Low Gray Level Run Emphasis	-0.1428	(0.6114)	0.8464	***(<0.0001)***	0.0642	(0.8224)	0.0964	(0.7337)	-0.0357	(0.9030)	0.3178	(0.2479)
Gray Level Run Length Matrix High Gray Level Run Emphasis	0.0964	(0.7337)	-0.8964	***(<0.0001)***	-0.0107	(0.9744)	-0.2178	(0.4342)	0.05	(0.8625)	0.0178	(0.9540)

Table S3: Correlation between textural parameters and failure load for the femoral neck and the great trochanter in CT images.

In bold and italic, p-value under 0,05. (aBMD : areal bone mineral density ; GLCM : grey level coocurrence matrix ; GLRM : grey level run-length matrix).

	Neck		GT	
**Textural Parameters**	r	p value	r	p value
**First Order**				
Energy	0.010	(0.974)	0.371	(0.173)
Entropy	0.025	(0.933)	-0.057	(0.842)
Mean	-0.446	(0.097)	0.285	(0.301)
Median	-0.337	(0.218)	0.236	(0.396)
**GLCM**				
Contrast	0.242	(0.381)	0.035	(0.903)
Correlation	-0.307	(0.264)	0.053	(0.852)
Joint Energy	-0.092	(0.743)	-0.12	(0.657)
Joint Entropy	0.110	(0.695)	0.028	(0.923)
Inverse Difference Moment	-0.139	(0.620)	-0.167	(0.549)
Maximum Probability	-0.067	(0.812)	-0.157	(0.575)
Sum Average	-0.192	(0.49)	0.05	(0.862)
Sum of Squares	0.017	(0.954)	-0.057	(0.842)
**GLRM**				
Short Run Emphasis	0.214	(0.442)	0.125	(0.657)
Long Run Emphasis	-0.214	(0.442)	-0.107	(0.704)
Gray Level Non Uniformity	0.396	(0.144)	0.375	(0.169)
Run Length Non Uniformity	0.528	***(0.045)***	0.564	***(0.031)***
Run Percentage	0.214	(0.442)	0.146	(0.602)
Low Gray Level Run Emphasis	0.096	(0.733)	-0.035	(0.903)
High Gray Level Run Emphasis	-0.217	(0.434)	0.05	(0.862)

Table S4: Correlation between textural parameters and aBMD.
In bold and italic, p-value under 0,05. GT= greater trochanter.

Textural parameters	MRI Neck		MRI GT		MRI Inter trochanteric		CT Neck		CT GT		CT Inter trochanteric	
First Order Energy	0.0858	(0.7609)	-0.1377	(0.6244)	0.3577	(0.1904)	-0.1605	(0.5525)	0.1914	(0.4775)	0.2415	(0.3674)
First Order Entropy	-0.2593	(0.3505)	-0.6314	***(0.0115)***	0.0697	(0.8048)	-0.3681	(0.1605)	0.0913	(0.7366)	-0.0854	(0.7531)
First Order Mean	0.0697	(0.8048)	-0.1162	(0.6798)	0.3434	(0.2100)	-0.7201	***(0.0016)***	0.2636	(0.3238)	0.1384	(0.6091)
First Order Median	0.0859	(0.7607)	-0.1341	(0.6335)	0.3112	(0.2587)	-0.5460	***(0.0286)***	0.2957	(0.2661)	0.1060	(0.6959)
Gray Level Co-occurrence Matrix Contrast	-0.2629	(0.3436)	-0.1359	(0.6290)	-0.2057	(0.4619)	0.1089	(0.6878)	0.0589	(0.8284)	-0.1590	(0.5562)
Gray Level Co-occurrence Matrix Correlation	0.1144	(0.6845)	-0.2504	(0.3679)	0.3094	(0.2616)	-0.7643	***(0.0005)***	0.2577	(0.3351)	0.1222	(0.6519)
Gray Level Co-occurrence Matrix Joint Energy	0.2880	(0.2978)	0.5992	***(0.0182)***	0.0322	(0.9093)	0.3622	(0.1678)	-0.0810	(0.7655)	0.1575	(0.5599)
Gray Level Co-occurrence Matrix Joint Entropy	-0.3273	(0.2336)	-0.5831	***(0.0224)***	-0.0053	(0.9848)	-0.2886	(0.2782)	0.0427	(0.8752)	-0.1516	(0.5749)
Gray Level Co-occurrence Matrix Inverse Difference Moment	0.1645	(0.5577)	0.1806	(0.5193)	0.4025	(0.1369)	0.1163	(0.6678)	-0.1030	(0.7039)	0.2739	(0.3045)
Gray Level Co-occurrence Matrix Maximum Probability	0.1824	(0.5151)	0.5670	***(0.0274)***	0.0143	(0.9596)	0.2886	(0.2782)	-0.2768	(0.2992)	0.1634	(0.5452)
Gray Level Co-occurrence Matrix Sum Average	-0.1610	(0.5664)	-0.5330	***(0.0407)***	0.0572	(0.8394)	-0.3195	(0.2275)	0.1870	(0.4879)	-0.3888	(0.1366)
Gray Level Co-occurrence Matrix Sum of Squares	-0.2182	(0.4345)	-0.5563	***(0.0312)***	0.0483	(0.8642)	-0.3799	(0.1465)	0.1222	(0.6519)	-0.1413	(0.6014)
Gray Level Run Length Matrix Short Run Emphasis	-0.2128	(0.4462)	-0.1914	(0.4943)	-0.2540	(0.3609)	0.0810	(0.7655)	0.1089	(0.6878)	-0.2577	(0.3351)
Gray Level Run Length Matrix Long Run Emphasis	0.1645	(0.5577)	0.1359	(0.6290)	0.2880	(0.2978)	-0.1089	(0.6878)	-0.1281	(0.6362)	0.2577	(0.3351)
Gray Level Run Length Matrix Gray Level Non Uniformity	-0.1717	(0.5405)	0.3327	(0.2255)	0.0536	(0.8493)	0.4742	(0.0634)	0.0854	(0.7531)	0.2430	(0.3644)
Gray Level Run Length Matrix Run Length Non Uniformity	-0.2683	(0.3335)	-0.0715	(0.7999)	-0.0715	(0.7999)	0.3078	(0.2461)	0.1826	(0.4984)	-0.0810	(0.7655)
Gray Level Run Length Matrix Run Percentage	-0.1717	(0.5405)	-0.1359	(0.6290)	-0.2307	(0.4079)	0.0810	(0.7655)	0.1251	(0.6441)	-0.2577	(0.3351)
Gray Level Run Length Matrix Low Gray Level Run Emphasis	0.0858	(0.7609)	0.5330	***(0.0407)***	-0.0447	(0.8742)	0.5228	***(0.0377)***	-0.1104	(0.6838)	0.3063	(0.2485)
Gray Level Run Length Matrix High Gray Level Run Emphasis	-0.1806	(0.5193)	-0.5295	***(0.0423)***	0.0608	(0.8295)	-0.3431	(0.1931)	0.2150	(0.4238)	-0.3873	(0.1382)
